# A consensus-based evaluation framework for telehealth in resource-constrained settings: evidence from a community Delphi study in Colombia

**DOI:** 10.3389/fdgth.2026.1803163

**Published:** 2026-07-06

**Authors:** John Michael Diaz, Ysabel Polanco, Lara Vargas Alvarez, Camila Penso, Ashley Cardona, Juan Fernando Mesa

**Affiliations:** 1 Department of Agricultural Education and Communication, University of Florida, Gainesville, FL, United States; 2 Faculty of Medicine, Universidad de Antioquia, Medellín, Colombia

**Keywords:** Colombia, community health workers, community-based participatory research, Delphi method, digital health, health equity, telehealth, telemedicine evaluation

## Abstract

**Introduction:**

Telehealth has expanded rapidly as a strategy to improve access to health care for underserved populations, yet robust, equity-focused evaluation frameworks remain limited in low-resource and displacement-affected settings. In Colombia, rural and peri-urban communities such as Granizal face persistent geographic, financial, and infrastructural barriers to care despite formal insurance coverage. Community-informed approaches are needed to ensure that telehealth programs are responsive, effective, and sustainable.

**Methods:**

This study employed a three-round Delphi methodology to develop a consensus-based evaluation framework for a community telehealth program in Granizal, Antioquia. Fifteen panelists representing academic researchers, community health workers (promotoras de salud), and municipal decision-makers participated across all rounds. The panelists were selected and included according to their experience and expertise with telehealth services and also based on their community involvement specifically in Granizal. Phase 1 used open-ended questionnaires to elicit relevant data elements, barriers, engagement strategies, and access solutions. Responses were analyzed using thematic analysis. In Phase 2, panelists rated identified items using a five-point Likert scale. Phase 3 prioritized items meeting predefined consensus thresholds. Ethical approval was obtained from institutional review boards in Colombia and the United States.

**Results:**

Consensus was achieved across seven evaluation domains: pre-visit data, post-visit data, follow-up data, health post resources, barriers, community engagement strategies, and access solutions. Panelists prioritized comprehensive clinical and sociodemographic data, continuity of care indicators, patient experience measures, and follow-up outcomes related to adherence and quality of life. Key barriers included limited connectivity, insufficient infrastructure, fragmented insurance coverage, workforce instability, and lack of political commitment. CHWs were identified as central to patient engagement, digital literacy support, and program sustainability. The resulting framework integrates clinical, social, infrastructural, and behavioral indicators aligned with local realities.

**Conclusions:**

This study demonstrates the value of participatory, consensus-based approaches for designing telehealth evaluation frameworks in underserved settings. By embedding community health workers and local stakeholders in the evaluation process, the framework advances equity, contextual relevance, and implementation feasibility. The Granizal model offers a replicable approach for strengthening telehealth evaluation and decision-making in similar low-resource and displacement-affected communities.

## 1 Introduction

Persistent inequities in access to quality healthcare continue to affect marginalized populations worldwide, including refugees, internally displaced persons, and rural residents who face geographic, infrastructural, and financial barriers to care (1–3). Telehealth has emerged as a strategy to mitigate these disparities by reducing travel burdens, expanding access to clinical expertise, and supporting continuity of care in settings where traditional health services are limited (4, 5). Yet the benefits of telehealth remain unevenly distributed. Stable connectivity, functional devices, digital literacy, and clear regulatory frameworks are essential for effective implementation, and these conditions are inconsistently met across low-resource regions (6–8).

Across Latin America, telehealth has helped alleviate hospital overcrowding and improve chronic disease management, but high device costs, variable broadband penetration, and fragmented regulatory environments continue to restrict equitable adoption (5, 6). These challenges are particularly pronounced in Colombia, where the national health system aims for universal coverage but structural inequities persist between urban and rural areas. Rural and peri-urban communities often lack local health centers, reliable transportation, and diagnostic capacity, leaving formally insured households effectively isolated from routine care (9–11). Although telemedicine use increased substantially during the COVID-19 pandemic, accounting for nearly one fifth of all healthcare contacts by 2020 (12, 13), access remains shaped by socioeconomic divides. Patients in the subsidized insurance regime experience greater reductions in service utilization and face more pronounced digital barriers than those in the contributory regime (14).

Despite these constraints, Colombian evidence demonstrates that telehealth can strengthen the health system when programs incorporate community feedback, provide adequate training, and are supported by robust infrastructure (7, 15, 16). However, long-term adoption remains hindered by inconsistent governmental investment, uneven broadband expansion, and the absence of evaluation frameworks that reflect the realities of underserved communities, including those affected by displacement (1, 16). This gap is especially relevant in Granizal, a peri-urban settlement with a long history of social and infrastructural marginalization, where residents routinely encounter geographic, financial, and administrative barriers to care. Granizal's combination of displacement, informality, and limited health system integration makes it a critical case for developing context-sensitive telehealth evaluation tools.

### 1.1 Community-engaged approaches to telehealth evaluation

Community engagement is increasingly recognized as essential for designing equitable digital health interventions. Extension systems, which are collaborative networks linking universities, government agencies, nonprofits, and community members, offer a structured mechanism for co-developing and evaluating telehealth programs in ways that reflect local priorities and sociocultural contexts (17, 18). These systems help ensure that evaluation frameworks capture barriers such as digital literacy gaps, connectivity limitations, and regulatory constraints that may otherwise be overlooked (19, 20). In rural and resource-constrained settings, systematic evaluation is critical for demonstrating value, guiding investment, and ensuring that telehealth programs remain responsive to community needs (18, 20).

Despite the rapid expansion of telehealth, participatory evaluation frameworks remain scarce, particularly in communities affected by displacement or chronic social vulnerability. These populations often face unstable living conditions, limited digital access, and fragmented continuity of care, yet few evaluation models account for these intersecting challenges (21). Evidence from delivery science underscores the value of participatory approaches that embed community health workers and local stakeholders as operational anchors, improving program relevance, feasibility, and sustainability (22–24). Embedding community-driven consensus indicators within evaluation designs is therefore essential for ensuring that digital health interventions remain empirically viable and responsive to the lived realities of vulnerable populations.

### 1.2 Purpose and objectives

This study was undertaken to design, evaluate, and continuously refine a telehealth program for Granizal, an urban district in Antioquia where residents routinely face social, geographic, and financial barriers that limit access to quality care. By establishing a rigorously grounded evaluation framework, the study aims to provide local government agencies, university partners, and community stakeholders with the tools necessary to implement, monitor, and improve telehealth services in a way that advances health equity.

The specific objectives included:

- Determine whether a consensus can be reached among experts and community members on the core components and evaluation methodology for Granizal's telehealth program.
- Identify and prioritize the data elements and metrics most appropriate for assessing program effectiveness.
- Detect barriers that impede broader community participation in telemedicine services.
- Explore strategies to engage Granizal residents with telehealth offerings.
- Recognize solutions that enhance access and sustain telemedicine services in the community.

Panelists including academic researchers, government officials, nonprofit representatives, and community members provided structured feedback on proposed evaluation components, anticipated challenges, and potential successes. Using the Delphi method, each component was ranked on a Likert scale from least to most important to achieve consensus. The resulting prioritization informs the design, monitoring, and iterative improvement of the telehealth program.

The findings will inform policy and resource allocation decisions, ensuring that future telehealth initiatives in Granizal are efficient, sustainable, and aligned with local needs. By foregrounding community knowledge alongside provider expertise, this study offers an innovative, community-based model for telehealth implementation that can be adapted to other urban Colombian contexts facing similar challenges.

## 2 Materials and methods

### 2.1 Research strategy

This study employed a three-round Delphi technique, an iterative process using anonymous questionnaires to build expert consensus within a short timeframe (25, 26). In community settings, the method promotes open communication, mitigates individual biases, and fosters collective decision making (27). Early anonymity limits the influence of dominant voices, encouraging diverse perspectives (28, 29), while subsequent rounds allow participants to refine their views in response to group feedback, leading to more informed consensus. In public health and community development, the Delphi approach serves as an effective platform for identifying shared goals, priorities, and strategies critical for engagement and sustainable action (25, 30, 31). Its iterative nature enables exploration of complex issues from multiple perspectives, strengthening agreement, shared ownership, and participant commitment (32).

### 2.2 Delphi panel selection and composition

The present investigation was carried out in the Department of Antioquia, a region with a growing demand for telehealth services that remains understudied. Fieldwork focused on the informal settlement of Granizal, located within the municipality of Bello between the boundaries of Medellín and Copacabana, where residents experience persistent social, geographic, and financial barriers to healthcare. To embed local perspectives, panelists were recruited through a community-based participatory research approach guided by recommendations from Granizal's neighborhood leaders and local nonprofit organizations. Purposive selection criteria were based on stakeholders’ professional experience, community involvement, and telehealth expertise. Recruitment involved initial consultative meetings with community leaders to identify eligible candidates, followed by formal invitations to fifteen experts whose experience ranged from five to thirty years.

The multidisciplinary Delphi panel comprised fifteen experts drawn from the three pillars that sustain Granizal's telehealth initiative: seven academics from the University of Antioquia, five community health workers (promotoras de salud), and three municipal decision makers, as detailed in Table 1. Academic researchers contributed biomedical and health systems expertise through their work with the University of Antioquia's Digital Hospital platform. Community health workers were included for their direct involvement in telehealth screening and public health activities within Granizal, providing culturally grounded operational insight. Municipal representatives were selected for their administrative oversight of telehealth and care coordination within the regional health system. Together, this balanced panel supplied the technical, operational, and administrative perspectives needed to evaluate a telehealth model responsive to the needs of Granizal residents.

**Table 1:** Panel composition and characteristics.

Panelist	Professional Role	Disciplinary background	Practice or service setting	Years of Experience	Area of Expertise
1	Academic	Medical doctor and Masters in Public health	University of Antioquia	30	Professor Public health Health administration
2	Academic	Medical doctor and Masters in Public health	University of Antioquia	22	Professor Public health
3	Academic	Environmental engineer and Masters in Public health	University of Antioquia	20	Professor Public health
4	Academic	Medical doctor and Masters in Public health	University of Antioquia	18	Professor Public health. Health administration
5	Academic	Nurse and Masters in Telehealth	University of Antioquia	10	Digital hospital Coordinator
6	Academic	Medical doctor and Masters in Telehealth	University of Antioquia	8	Digital Hospital Director
7	Academic	Medical doctor	University of Antioquia	5	Digital hospital Assistant
8	Community Health Worker	Public Health Technician	Health center Granizal	25	Telehealth and health screening
9	Community Health Worker	Public Health Technician	Health center Granizal	22	Telehealth and health screening
10	Community Health Worker	Public Health Technician	Health center Granizal	18	Telehealth and health screening
11	Community Health Worker	Public Health Technician	Health center Granizal	16	Health screening
12	Community Health Worker	Public Health Technician	Health center Granizal	16	Health screening
13	Municipal health representative	Medical doctor	Health Insurance Company Bello Salud	17	Assistant manager Bellosalud
14	Municipal health representative	Medical doctor	Health Insurance Company Bello Salud	12	Medical director telehealth Bellosalud
15	Municipal health representative	Computer engineer	Antioquia Health Department	8	Telehealth computer engineer coordinator

The selection of fifteen panelists was methodologically guided by the localized scope of the inquiry and the high degree of shared expertise among participants. Delphi methodology emphasizes that panel size is determined by the specificity of the topic and the homogeneity of expertise rather than statistical power, and studies with ten to fifteen participants are considered sufficient to generate stable consensus in focused health systems research (33). Keeping the panel within this range also aligns with contemporary guidance recommending eight to twenty participants to reduce attrition and maintain depth of qualitative input (34).

To ensure that panel heterogeneity did not compromise consensus stability, we followed established Delphi guidance supporting the inclusion of diverse stakeholder groups when anonymity and structured iteration are used to prevent dominance effects (Hsu and Sandford, 2007; Keeney et al., 2011; Jünger et al., 2017). Stability was assessed by examining whether items showed meaningful shifts in agreement across rounds. After the second round, no substantial changes were observed in the proportion of panelists rating items as important or very important, which is an accepted indicator of response stability in Delphi studies that do not employ distributional statistics (35). Open-ended comments were also reviewed to determine whether new concepts continued to emerge. Consistent with de Villiers et al. (2005), the absence of new themes indicated that conceptual saturation had been reached. Representation across stakeholder groups was ensured through purposive sampling and by maintaining full participation of all three groups throughout the rounds.

An implementation workgroup was established as a voluntary subgroup of the Delphi panel for post-consensus activities. Membership was open to all panelists and based solely on willingness to support local implementation. To avoid influencing the analytic process, the workgroup convened only after consensus was reached and focused exclusively on applying the framework, including coordinating training for community health workers, adapting data tools, and facilitating communication between institutional partners and neighborhood stakeholders.

### 2.3 Study context

This study took place in Granizal, a rural division in the municipality of Bello, located in the Department of Antioquia, northwestern Colombia. Founded in 1995, it has grown into the region's second largest informal settlement, housing more than 22 000 residents most of whom are Internally Displaced Population (IDP) who arrived from Antioquia and neighboring departments such as Chocó, Caldas, and Quindío (13). The community has faced constrained governmental support, land tenure ambiguities, and periodic social unrest, yet it has remained determined to reconstruct essential services and pursue socioeconomic development. In the past decade, residents have rebuilt housing, roads, markets, agricultural terraces, sanitation and water systems, primary health facilities, and schools (13). The settlement's collective effort has transformed a once vacant territory into an organized community that prioritizes peace, the rule of law, and shared prosperity (1, 13).

A single health post now serves Granizal. Established through a partnership with TECHO Colombia, Children Beyond Our Borders, CrediCorp Capital, and a cadre of dedicated volunteers, the post houses eight patient beds, a waiting lobby, a bathroom, and a private room. Recent upgrades have added a television and a laptop, equipping the facility to conduct telehealth consultations. Central to the post's operation are the CHWs known locally as *Promotoras de Salud* (also called *Lideresas*). These women often lack formal medical credentials. However, few of them have received the degree of public health technicians. They are highly trusted members of the community who bridge the gap between formal practitioners and residents, deliver preventive education, and advocate for health seeking behaviors. Their unheralded contribution is especially critical in a setting where formal health professional density is low. Integrating CHWs into any telehealth design is therefore essential for attaining community acceptance, facilitating data collection, and ensuring that care plans are culturally consonant.

The converging realities of geographic isolation, perennial infrastructure deficits, economic hardship, and a resilient, community driven population render Granizal a natural laboratory for testing a multidisciplinary telehealth intervention. By drawing on the University of Antioquia's clinical expertise and digital health platform (The Living Lab Telesalud “Digital Hospital”), integrating the operational oversight of the municipal health administration, and leveraging the trusted touchpoints of CHWs, the study seeks to create an evaluation framework that is both evidence-based and locally relevant. The resulting model aspires to expand access, reduce inequities, and lay a scalable foundation for future telehealth initiatives in Granizal and also in similar resource constrained settings where health services needs are very high, transportation is difficult and there are limited health personnel and health centers to provide adequate health services.

### 2.4 Data collection and analysis

Consistent with established Delphi methodology (30) the study unfolded in three successive phases from fall 2024 through spring 2025. All correspondence was conducted in Spanish to accommodate the linguistic preferences of the panelists and to minimize translation-related bias. Phase 1 focused on rapid consensus building, an approach chosen to respect the limited availability of the CHWs and the heightened stress experienced by the community following a recent landslide. In Phase 1, panelists completed an open-ended questionnaire consisting of seven targeted prompts. Panelists were asked to list the patient data or information that should be collected before the telehealth visit, immediately after the telehealth visit, and during a follow-up call to ensure a comprehensive evaluation of the telehealth program. Additionally, they were asked to list the resources the Granizal health center needs to effectively meet patient needs, the most significant barriers, obstacles, or strategies for engaging the Granizal community with telehealth services, and all potential solutions to improve both access and community engagement in telehealth services in Granizal, Medellín.

The qualitative data were analyzed using Braun and Clarke's six-step thematic analysis framework (36). Two co-authors independently familiarized themselves with the transcripts, extracted relevant text segments from the results, and generated initial open codes. To ensure researcher validation and inter-coder reliability, the authors cross-examined the independent codebooks. Discrepancies regarding code definitions or application were resolved through iterative dialogue and consensus meetings until a single, unified coding structure was achieved. The analysis was conducted manually without the use of specialized qualitative software; instead, the research team used a structured Excel spreadsheet to organize codes, track revisions, and document analytic decisions. To enhance analytic credibility, we incorporated several formal procedures, including independent coding, iterative discrepancy resolution, and peer debriefing, consistent with established qualitative trustworthiness practices [Lincoln & Guba, 1985 (36);; Nowell et al., 2017]. Peer debriefing sessions with the broader research team and an external evaluator served as an additional credibility check, and coding decisions were advanced only when consensus was reached. Next, the authors systematically grouped these refined codes into provisional themes and categories, including barriers, strategies, and solutions, based on code recurrence and direct alignment with the study objectives. These provisional themes were further reviewed, merged, and divided into subthemes to ensure mutual exclusivity and internal homogeneity. Subsequently, a peer-reviewed draft of the thematic findings was circulated to the broader research team and an independent external evaluator for final validation, with revisions continuing until the team converged on the definitive set of themes used to populate the subsequent Delphi rounds.

Phase 2 incorporated the themes derived in Phase 1 and presented them to the same panel. Participants rated the importance of each identified data element, resource, barrier, strategy, and solution on a five-point Likert scale ranging from one for strongly disagree to five for strongly agree. To preserve the depth of community input while maintaining scientific rigor, the consensus criteria were structured around two distinct thresholds established *a priori* prior to the initiation of data collection. The use of differentiated consensus benchmarks across progressive rounds accommodates the shifting operational goals of each study phase, aligning with systematic reviews demonstrating that threshold variation is an established methodological practice within biomedical and health systems Delphi research (35, 37). During this initial phase of data synthesis, a more inclusive consensus threshold of sixty-six percent agreement was utilized; this baseline was intentionally selected to prevent the premature exclusion of novel indicators and ensure that broad, context-specific insights from community stakeholders were retained for further evaluation. Each item was followed by an open-ended prompt that invited additional suggestions.

In Phase 3, panelists prioritized the items that had reached consensus in Phase 2. During this final refinement phase, a more rigorous threshold of seventy-three percent agreement, representing eleven or more of the fifteen participants, was enforced to guarantee that the final evaluation framework was comprised exclusively of indicators achieving stable, high-level verification among the panelists. This staged approach allowed the instrument to remain highly sensitive to localized community healthcare realities during early iterations while ensuring definitive, methodologically robust consensus at the conclusion of the study (see Table 2). The fifteen panelists, consisting of seven academics from the University of Antioquia, five CHWs, and three municipal health representatives, remained active across all three phases and completed all surveys.

**Table 2:** Consensus criteria applied across delphi phases.

Phase	Purpose of Phase	Consensus Criterion Applied	Operational Definition	Justification
Phase 1 (Open-ended elicitation)	Generate comprehensive lists of data elements, resources, barriers, strategies, and solutions	No numerical threshold applied	All qualitative responses were retained and coded using thematic analysis	Early Delphi rounds are exploratory. Retaining all responses prevents premature exclusion of community-generated concepts and aligns with qualitative Delphi guidance.
Phase 2 (Importance rating)	Evaluate the importance of all items generated in Phase 1	**66 percent agreement**	An item advanced if at least two thirds of panelists rated it as important or very important	A more inclusive threshold in early rating rounds prevents the loss of novel or context-specific indicators. Systematic reviews show that threshold variation across rounds is an accepted Delphi practice (35, 37).
Phase 3 (Prioritization and final selection)	Prioritize items that reached consensus in Phase 2	**73 percent agreement** (11 of 15 panelists)	An item was retained only if at least 73 percent of panelists rated it as important or very important	A higher threshold in the final round ensures that the final framework reflects strong, stable agreement. The 70–80 percent range is widely used in health sciences Delphi studies.
Stability Check	Confirm reliability of consensus across rounds	No meaningful change in agreement between Phase 2 and Phase 3	Items were considered stable if the proportion of panelists rating them as important or very important did not shift substantially	Stability of agreement is an accepted indicator of consensus reliability in Delphi studies that do not use distributional statistics.
Saturation Check	Ensure completeness of concepts	No new concepts emerging in Phase 2 open-ended comments	Open-ended responses were reviewed to determine whether new themes appeared	The absence of new concepts indicates conceptual saturation, consistent with qualitative Delphi methodology (de Villiers et al., 2005).

### 2.5 Human ethics and consent to participate

This study was approved by the University of Florida Institutional Review Board (protocol #202400895) and the Ethics Committee of the University of Antioquia (document #069, 27 June 2024). All participants provided written informed consent prior to participation in this study. All procedures were conducted in accordance with relevant ethical guidelines and regulations.

### 2.6 Study limitations

While Delphi is valuable for eliciting expert consensus, the composition and perspectives of the panel may bias the outcome. Expert judgments may not fully capture the views of the broader community, especially those who were unable or unwilling to participate. Moreover, the exclusive focus on Granizal within the municipality of Bello limits the generalizability of the findings to other geographic or sociocultural contexts. Although a community-based participatory strategy was employed to select panelists, this process may have introduced selection bias if certain community voices were over- or underrepresented.

In addition, the study did not incorporate several quantitative rigor indicators commonly used in Delphi research, such as median values, interquartile ranges, or other measures of dispersion. The analytic strategy was defined *a priori* around percentage-agreement thresholds and a staged consensus process, which are accepted approaches within Delphi methodology. However, the absence of distributional statistics limits the extent to which the robustness of consensus can be evaluated using additional quantitative metrics. Future Delphi studies in similar settings would benefit from integrating these indicators to enhance methodological rigor and comparability.

### 2.7 Reflexivity statement

The research team recognizes that their positionality as external researchers affiliated with well-resourced academic institutions inevitably influenced the structural design, data interpretation, and thematic analysis of this study. Operating within an international land-grant and extension framework, the investigators held prior assumptions regarding the role of formal institutional networks, the definition of digital literacy, and what constitutes a successful health intervention. The team maintained a conscious awareness that these academic and administrative backgrounds could inadvertently introduce paternalistic biases or overshadow the localized insights of a community deeply affected by forced displacement and systemic exclusion. To actively mitigate these power asymmetries, the study design purposefully rejected a top-down extractive research model, utilizing instead a collaborative Delphi process that positioned local expertise as the primary arbiter of valid metrics.

Throughout the project, the researchers engaged in structured, iterative dialogue to challenge their own preconceptions regarding telehealth readiness and technology adoption. During the qualitative coding and category consolidation phases, positionality was explicitly questioned by cross-checking researcher-generated themes against the direct testimony of the field team. By integrating CHWs as authentic co-designers and evaluators rather than passive data collectors, the team structurally decentralized academic authority over the evaluation framework. This deliberate integration ensured that the final indicators directly reflected the operational realities and informal care networks of Granizal, rather than external institutional assumptions. This continuous commitment to methodological transparency and critical self-awareness minimized researcher-centric bias, safeguarded the authenticity of participant voices, and demonstrated the rigorous ethical accountability required when conducting research alongside socially vulnerable populations.

## 3 Results

**Figure 1:**
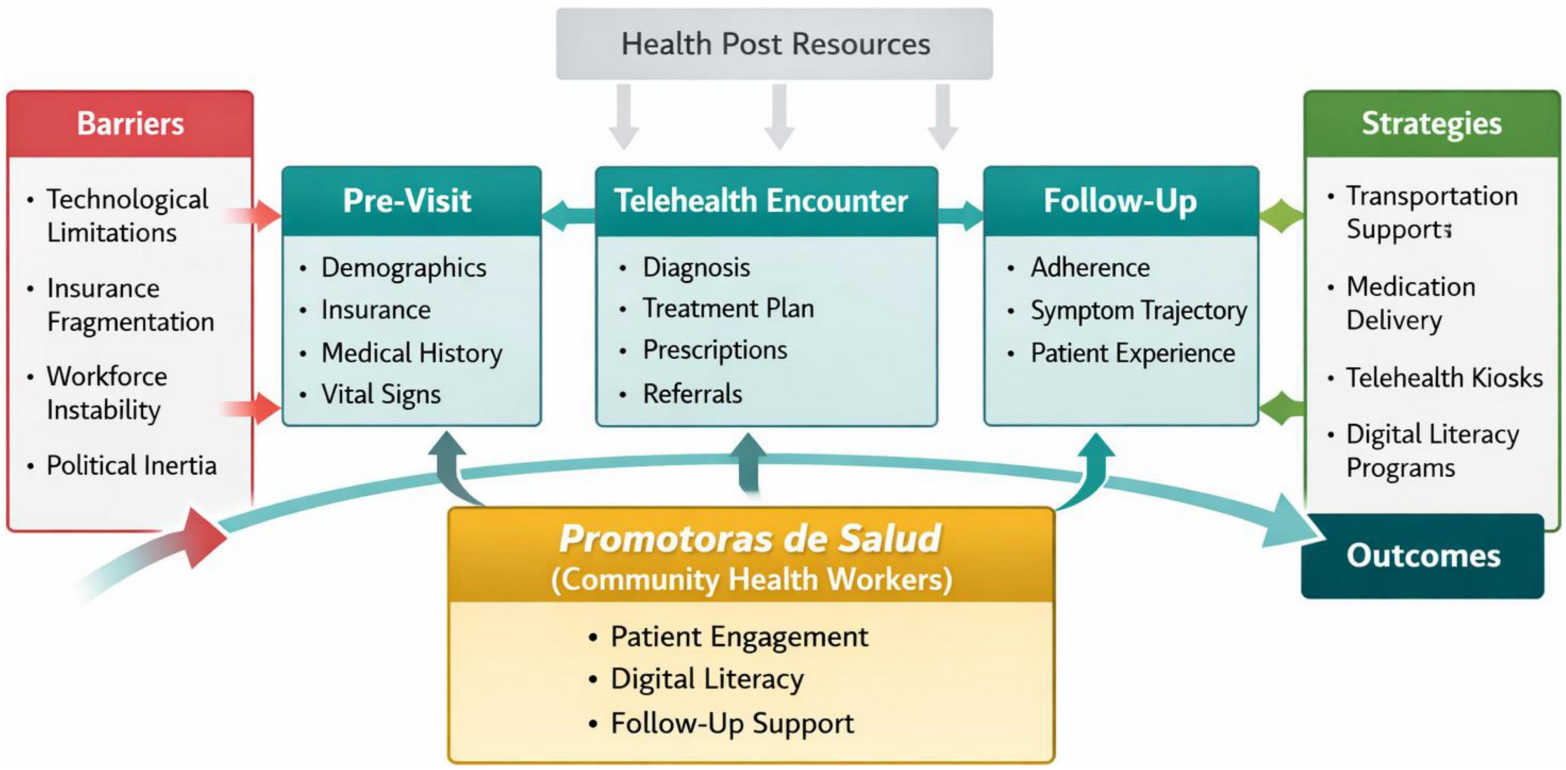
Consensus-based telehealth evaluation framework derived From delphi panel findings.

The Delphi panel yielded a consensus-driven, domain-based evaluation framework for the telehealth initiative in Granizal. Consensus was defined as seventy-three percent or greater combined agreement (agree or strongly agree) among the fifteen panelists. The final framework encompasses seven distinct evaluation domains: pre-visit data, post-visit data, follow-up data, health-post resources, implementation barriers, community engagement strategies, and localized access solutions. Figure 1 provides an integrated conceptual model that visually synthesizes the structural relationships between these consensus-derived domains.

### 3.1 Pre-visit, post-visit, and follow-up evaluation domains

For the pre-visit data domain, the panel prioritized baseline clinical and basic demographic inputs as critical prerequisites for initial screening assessments (Table 3). High consensus was achieved for collecting primary contact information, insurance provider names, formal informed consent, and core vital signs. Broad clinical parameters, including the main reason for consultation, current illness history, and previous treatments, were established as essential baseline variables. Qualitative responses emphasized that capturing these data points early serves as a clinical benchmark, allowing coordinators to evaluate changes in patient health status across the care continuum.

**Table 3:** Panel agreement on the importance of specific data elements to collect before a telehealth visit for comprehensive program evaluation.

Response	Agree	Strongly Agree	Both Agree and Strongly Agree
Personal, contact, and sociodemographic information (including place of residency), and companion^a^	13.3 (2)	86.6 (13)	100 (15)
Name of health insurance provider^a^	13.3 (2)	86.6 (13)	100 (15)
Informed consent (for care and follow-up) ^a^	20 (3)	80 (12)	100 (15)
Patient's access to basic sanitation^a^	33.3 (5)	53.3 (8)	86.6 (13)
Updated digital medical record^a^	40 (6)	40 (6)	80 (12)
Questions regarding mental health^a^	20 (3)	60 (9)	80 (12)
Reason for consultation and current illness^a^	6.6 (1)	93.3 (14)	100 (15)
Vital signs^a^	20 (3)	73.3 (11)	93.3 (14)
Identification of caregiver need^a^	26.6 (4)	46.6 (7)	73.3 (11)
Patient's access to information technology^a^	13.3 (2)	80 (12)	93.3 (14)
Digital literacy	20 (3)	46.6 (7)	66.6 (10)
Patient perception of telehealth^a^	33.3 (5)	53.3 (8)	86.6 (13)
Evaluation of need to improve the program of telehealth^a^	20 (3)	60 (9)	80 (12)
Previous treatments, exams, diagnoses and current related to the reason of the visit^a^	13.3 (2)	80 (12)	93.3 (14)
Ability to comply with follow-up or treatment^a^	20 (3)	46.6 (7)	66.6 (10)
Any pending appointments with specialists or diagnostic needs^a^	33.3 (5)	53.3 (8)	86.6 (13)
Family medical history^a^	26.6 (4)	60 (9)	86.6 (13)

a >73% consensus.

The post-visit data domain achieved complete consensus across all twelve proposed indicators, representing the highest level of absolute agreement within the data-collection parameters (Table 4). The panel prioritized tangible, action-oriented tracking variables that document the immediate resolution of the virtual encounter. These variables include the verification of a completed treatment plan, the issuance of clear diagnostic guidance, and the successful delivery of medical prescriptions. Panelists uniformly prioritized tracking care continuity metrics, specifically monitoring whether a patient requires immediate referral to higher-level healthcare facilities for specialized imaging, laboratory tests, or hospitalization.

**Table 4:** Panel agreement on the importance of specific data elements to collect after a telehealth visit for comprehensive program evaluation.

Response	Agree	Strongly Agree	Both Agree and Strongly Agree
Treatment plan^a^	13.3 (2)	86.6 (13)	100 (15)
The patient received a diagnosis and guidance^a^	26.6 (4)	73.3 (11)	100 (15)
The patient received a prescription and medications^a^	26.6 (4)	73.3 (11)	100 (15)
The patient needs to be referred^a^	13.3 (2)	86.6 (13)	100 (15)
Need to ensure continuity of care with the same physician/professional^a^	13.3 (2)	86.6 (13)	100 (15)
The patient can access medical orders and medications^a^	20 (3)	80 (12)	100 (15)
Whether the patient felt heard/satisfied^a^	33.3 (5)	66.6 (10)	100 (15)
Evaluation/capacity for resolution^a^	33.3 (5)	53.3 (8)	86.6 (13)
Patient education and self-care^a^	40 (6)	60 (9)	100 (15)
Digital medical record in information systems^a^	40 (6)	60 (9)	100 (15)
Evaluate patient's perception of the telehealth visit before and after^a^	26.6 (4)	53.3 (8)	80 (12)
If the patient required referral to a higher-level care center to help with imaging and hospitalization^a^	20 (3)	73.3 (11)	93.3 (14)

a >73% consensus.

For the longitudinal evaluation of the program, the follow-up call domain established a clear data structure focused on clinical trajectories and care adherence (Table 5). High consensus was sustained for tracking post-consultation medical progress, the emergence of treatment complications, and the completion rate of prescribed laboratory exams. Indicators assessing patient-reported variables, such as perceived changes in quality of life, financial impacts of virtual care, and whether the patient felt a continuous sense of professional accompaniment, met the formal selection criteria. These parameters shift the monitoring architecture from isolated clinical encounters to a continuous tracking model.

**Table 5:** Panel agreement on the importance of specific data elements to collect during a follow-up call for comprehensive program evaluation.

Response	Agree	Strongly Agree	Both Agree and Strongly Agree
Patient's medical progress since the teleconsultation^a^	13.3 (2)	80 (12)	93.3 (14)
Whether the patient improved or had any complications or adverse reactions to treatment plan^a^	20 (3)	80 (12)	100 (15)
The prescribed exams could be performed^a^	13.3 (2)	80 (12)	93.3 (14)
The patient was able to follow recommendations received such as treatment plan, medications, diagnostics ^a^	26.6 (4)	73.3 (11)	100 (15)
Treatment adherence^a^	26.6 (4)	73.3 (11)	100 (15)
Patient's perception of changes in quality of life related to telehealth^a^	53.3 (8)	33.3 (5)	86.6 (13)
Financial impacts of telehealth on the patient^a^	40 (6)	40 (6)	80 (12)
Patient would recommend telehealth to others^a^	40 (6)	53.3 (8)	93.3 (14)
Patient's recommendations to improve the service of telehealth^a^	46.6 (7)	53.3 (8)	100 (15)
Effectiveness of care or treatment via telehealth^a^	33.3 (5)	66.6 (10)	100 (15)
Evaluation of patient satisfaction in telehealth with qualified professional and appropriated tools^a^	33.3 (5)	66.6 (10)	100 (15)
Ongoing evaluation of connectivity and patient digital literacy^a^	46.6 (7)	46.6 (7)	93.3 (14)
Whether patients want care with a doctor in person or just via telehealth^a^	26.6 (4)	73.3 (11)	100 (15)
Whether the patient feels accompanied and confident during care^a^	26.6 (4)	73.3 (11)	100 (15)

a >73% consensus.

### 3.2 Resource requirements and structural barriers

Infrastructure and human resource priorities identified by the panel reveal the material prerequisites required to maintain local telehealth operations (Table 6). The panel achieved complete consensus on the necessity of local government intervention to habilitate the health post through an adaptive extramural outreach model, linking the local center to an authorized external provider. Essential structural resources achieved high consensus, including stable internet connectivity, updated computer hardware, private consultation rooms, and the installation of large-capacity water storage tanks ranging from five hundred to one thousand liters to secure basic clinical sanitation.

**Table 6:** Panel agreement on the importance of resources needed by the granizal health center to effectively meet patient telehealth needs.

Response	Agree	Strongly Agree	Both Agree and Strongly Agree
Technology (internet, computers) ^a^	20 (3)	73.3 (11)	93.3 (14)
Privacy in consultation rooms^a^	40 (6)	60 (9)	100 (15)
Availability of more basic medical equipment for patient evaluation^a^	33.3 (5)	53.3 (8)	86.6 (13)
Supply of bedding (pillows, blankets, sheets) ^a^	20 (3)	40 (6)	60 (9)
Hygiene products (brooms, mops, soap, etc.) ^a^	40 (6)	46.6 (7)	86.6 (13)
More human resources (certified staff, nurses, health promotors) ^a^	13.3 (2)	80 (12)	93.3 (14)
Better physical infrastructure^a^	20 (3)	73.3 (11)	93.3 (14)
Coordination of activities between the community, academy, government to conduct health fairs and education^a^	13.3 (2)	80 (12)	93.3 (14)
Habilitate the health center by the local government to provide health services and telehealth^a^	13.3 (2)	86.6 (13)	100 (15)
Facilitating the delivery of medicines in the community^a^	6.6 (1)	93.3 (14)	100 (15)
Guarantee of drinking water (500–1,000-liter tanks) and electricity to establish the health center^a^	13.3 (2)	80 (12)	93.3 (14)
Commitment of health insurance providers to guarantee continuous and comprehensive care for the population^a^	6.6 (1)	93.3 (14)	100 (15)
Commitment of decision-makers^a^	6.6 (1)	93.3 (14)	100 (15)
Promote financial resources so that the community has an in-person interdisciplinary health team^a^	13.3 (2)	86.6 (13)	100 (15)
Start with an outreach service linked to a provider while the health center is being enabled^a^	6.6 (1)	86.6 (13)	93.3 (14)
Train more people in the health field to influence public health policies^a^	6.6 (1)	86.6 (13)	93.3 (14)

a >73% consensus.

These resource demands correspond directly with the prioritized implementation barriers outlined in Table 7. The panel demonstrated high consensus regarding severe environmental and institutional bottlenecks, including unstable territorial broadband capacity, physical difficulties contacting transient or displaced patients, and a systemic absence of major health insurance providers within the informal settlement. Structural barriers, such as rigid state licensing frameworks, a total lack of municipal sanitation infrastructure, and an acute deficit of trained medical personnel with long-term job stability, were rated as principal obstacles that restrict community engagement and undermine programmatic continuity.

**Table 7:** Panel agreement on the strategies to engage the granizal community with telehealth services.

Response	Agree	Strongly Agree	Both Agree and Strongly Agree
Greater adherence of staff to patient follow-up and monitoring^a^	26 (4)	66 (10)	100 (15)
More government commitment (providing economic, technological, and human resources) ^a^	7.1 (1)	92.8 (13)	93.3 (14)
Support Granizal so community health promoters can work in their own community^a^	14.2 (2)	85.7 (12)	93.3 (14)
More institutional support to establish the health center^a^	0	100 (12)	80 (12)

a >73% consensus.

### 3.3 Community engagement strategies and localized access solutions

The panel demonstrated strong agreement on a set of strategies to strengthen community engagement and operational stability for telehealth services in Granizal. As summarized in Table 7, participants reached unanimous consensus on the need for consistent staff adherence to patient follow-up and monitoring procedures, which reflects the central role of frontline clinical fidelity in telehealth operations.

Beyond micro-level clinical processes, the panel also prioritized institutional and community-embedded supports (Table 8). High consensus was achieved for expanding material and logistical resources for community health workers, enabling them to conduct health initiatives within their own neighborhoods. Participants also strongly supported increased municipal government commitments that include economic, technological, and human resources to reinforce the telehealth infrastructure. In addition, there was broad agreement on the importance of securing baseline institutional support to formally establish and stabilize the local health post facility.

**Table 8:** Panel agreement on the importance of addressing barriers affecting community engagement with telehealth services in granizal.

Response	Agree	Strongly Agree	Both Agree and Strongly Agree
Technological limitations and barriers^a^	21.4 (3)	78.6 (11)	93.3 (14)
Difficulty contacting patients^a^	35.7 (5)	64.3 (9)	93.3 (14)
Difficulty accessing appropriate treatment (medications, exams, diagnoses) ^a^	13.3 (2)	86.6 (13)	100 (15)
Medical services provided by health insurers are restricted and insufficient greater coordination and integration of health systems is required^a^	13.3 (2)	86.6 (13)	100 (15)
Many health insurers are absent in the territory, members don't have access to telehealth^a^	13.3 (2)	86.6 (13)	100 (15)
There are no sanitary conditions in the health center (drinking water and sewer system or septic tank), which prevents its authorization by the local health department^a^	23 (3)	76.9 (10)	86.6 (13)
There is a lack of sufficient, trained health personnel with job stability^a^	14.2 (2)	85.7 (12)	93.3 (14)
Lack of political will from decision-makers (governmental and institutional) to take action in the community^a^	0	100 (13)	86.6 (13)

a >73% consensus.

### 3.4 Evaluation of non-consensus items and stakeholder divergence

While the panel demonstrated strong overall convergence across the major domains, notable analytical variations and points of non-consensus emerged during the final phase, reflecting the distinct institutional positions of the participants. A primary divergence developed regarding pre-visit data requirements; community health workers (*promotoras de salud*) uniformly rated variables measuring patient digital literacy, home sanitation infrastructure, and broader psychosocial family histories as essential clinical precursors. Conversely, academic researchers and municipal decision-makers rated these elements significantly lower, preventing them from reaching the seventy-five percent consensus threshold. This variation indicates that frontline health workers prioritize localized social determinants of health that affect daily treatment compliance, whereas centralized stakeholders emphasize streamlined clinical throughput and traditional bio-centric data fields.

Divergence also emerged within the barrier and resource domains regarding personnel and administration. While all fifteen panelists recognized the general lack of trained medical personnel as a critical systemic bottleneck, the community health workers uniquely identified long-term job stability and local contract security as mandatory evaluation variables. Academic and municipal representatives, operating from institutional perspectives focused on macro-level resource allocation and budget management, prioritized short-term volunteer recruitment and rotating educational health fairs over structural labor integration. Furthermore, regarding administrative barriers, municipal representatives and academic experts prioritized compliance with state licensing guidelines like Resolution 3100, scoring traditional infrastructure adjustments as immediate imperatives. Frontline health workers, facing daily resource limitations, rejected these rigid timelines and instead achieved consensus around an adaptive, transitional strategy that deploys extramural outreach services via an external provider to maintain care continuity while long-term structural changes are pursued.

## 4 Discussion

The results of this Delphi study demonstrated substantial consensus among academics, municipal health representatives, and community health workers on the data elements, barriers, engagement strategies, and solutions needed to evaluate and strengthen telehealth implementation in Granizal. These findings highlight the interconnected nature of technical, social, and structural factors that shape access to virtual care in underserved communities. The following discussion situates these results within the broader telehealth and digital equity literature, compares the study's identified priorities to global frameworks, and interprets how these findings and the consensus-based framework derived from the Delphi process advance the science and practice of telehealth evaluation in Latin America.

However, given the exploratory and context-specific nature of this study, these findings represent an initial baseline rooted in the unique socio-political and structural realities of a single informal settlement. While the participatory methodology itself offers a transferable model for community-engaged digital health research, the specific metrics and priorities identified by this panel are deeply bound to local conditions and should not be assumed to possess immediate, universal applicability across other vulnerable or displaced populations without systematic adaptation and external validation.

### 4.1 Deconstructing the evaluation architecture: literature, innovation, and local context

To clearly define the scientific contribution of this study, the resulting evaluation framework must be understood as a multi-layered architecture comprising standard elements derived from existing literature, conceptual innovations in implementation science, and context-specific indicators emerging uniquely from the participatory process in Granizal.

The baseline layer of the framework incorporates standardized variables well-established in global digital health literature. The data elements prioritized by the panel, such as core clinical vital signs, primary demographic profiles, disease burden, and macro-level patient satisfaction metrics, directly align with several domains of the World Health Organization's Consolidated Telemedicine Implementation Guide (11, 13) and the National Quality Forum (NQF) telehealth measurement framework (25). These traditional metrics are essential for satisfying basic clinical reporting and institutional interoperability requirements (25, 38).

The primary conceptual novelty of this study lies in the structural elevation of community health workers (*promotoras de salud*) from passive program implementers to active co-designers and systems evaluators. While conventional digital health metrics predominantly focus on clinical throughput and technology uptime (17, 21), this framework explicitly integrates frontline health worker retention and workload data into the core evaluation design (24, 29). Treating these local actors as active evaluators captures crucial, often invisible dimensions of care delivery, such as localized digital literacy barriers and community trust dynamics, shifting the theoretical paradigm of telehealth monitoring toward sustainable, co-produced metrics (29, 39).

The most highly contextualized layer of the framework consists of specific variables that emerged uniquely from the participatory process conducted with the Granizal panel. These indicators directly reflect the Lived realities of internally displaced populations and informal settlement conditions. For example, the panel reached consensus on the necessity of systematically tracking patients’ forced displacement histories, local environmental shocks, the availability of informal caregiving networks, and household access to basic sanitation utilities. While global frameworks recognize access and experience conceptually (25, 40), they do not operationalize equity-focused measures at this granular level. The Granizal framework thus extends conventional models by converting abstract social determinants of health into measurable, actionable evaluation variables (40, 41).

### 4.2 Socio-digital barriers and the feasibility of framework implementation

A critical examination of the real-world feasibility of implementing this framework reveals substantial operational boundaries. While the panel successfully reached consensus on a comprehensive suite of evaluation metrics, the acute infrastructure limitations, institutional fragmentation, regulatory restrictions, and limited political commitment documented in the results present severe bottlenecks for data collection and programmatic sustainability.

Within this landscape of constraints, the panel's consensus on engagement strategies offers an important counterbalance rather than a complete solution. Prioritizing consistent staff adherence to follow-up protocols, expanding material and logistical support for community health workers, strengthening municipal commitments, and securing institutional backing for the health post reflects a recognition that telehealth implementation in Granizal requires both technical capacity and stable, community-embedded operational support. These priorities align with broader evidence that digital health initiatives in underserved settings depend on reliable frontline workforce engagement, institutional coordination, and locally grounded implementation structures (15, 26, 42). Similar to findings in other fragile or resource-limited contexts, the panel's emphasis on CHW support and municipal involvement underscores that telehealth sustainability is shaped as much by social and organizational infrastructure as by technological readiness (8, 18, 32). By identifying these engagement strategies as essential components of feasibility, the panel articulated pragmatic pathways that can help mitigate, although not fully resolve, the operational and institutional barriers documented in this study.

Despite these counterbalancing strategies, the digital divide remains a primary determinant of telehealth access and evaluation feasibility (4, 8). The framework requires continuous data collection, yet Granizal lacks stable territorial broadband capacity, and patients experience severe digital literacy barriers and high rates of residential instability, making long-term follow-up tracking logistically complex (8, 18). Furthermore, institutional fragmentation complicates the implementation of these metrics (10, 42). Many health insurance providers remain entirely absent from the territory, and the existing public health infrastructure lacks coordinated care mechanisms to easily integrate or aggregate qualitative, non-standardized community health inputs into regional databases (32, 42).

Regulatory restrictions add an additional layer of operational difficulty. Rigid state licensing frameworks, specifically compliance with standards such as Resolution 3100, present complex hurdles for localized health posts that lack basic sanitation utilities like municipal drinking water and sewer networks (43). Requiring strict adherence to these administrative and infrastructural demands before executing telehealth services could completely stall implementation in resource-constrained informal settlements (43, 44).

To navigate these real-world constraints, the feasibility of the framework relies on the adaptive, practical solutions prioritized by the panel. Rather than allowing rigid state regulations or infrastructure deficits to halt operations, the panel achieved consensus around an extramural outreach model. Linking the local health post directly to an already authorized external healthcare provider serves as a feasible, transitional pathway (44, 45). This strategy maintains legal compliance and operational continuity while long-term structural and utility adjustments are pursued.

Additionally, by embedding practical logistical metrics, such as medication delivery timeliness, transport availability, and the establishment of designated telehealth kiosks, the framework aligns its data collection demands with realistic, multi-modal access strategies that accommodate regional vulnerabilities rather than ignoring them (15, 45). From a human factors perspective, evaluating the *promotoras’* cognitive workload and role-mutation within this framework is critical (26, 46). Ensuring that the transitional extramural pathway balances clinical tracking with field-level workflow capacity is vital to prevent administrative overload and protect frontline workforce sustainability (26, 30).

### 4.3 Evaluating points of disagreement: where stakeholder priorities diverge

To achieve a rigorous analytical standard, the points of divergence within the Delphi panel must be evaluated as primary findings that reveal how institutional positions bound the consensus-building process. A critical analysis of the specific indicators and strategies that failed to achieve the mandatory seventy-three percent consensus threshold in Phase Three highlights deep structural tensions and differing perceptions regarding implementation feasibility among academics, municipal leaders, and frontline healthcare personnel.

A primary point of friction emerged around the proposed integration of extensive, real-time diagnostic reporting metrics within the telehealth screening architecture. This strategy was heavily advocated by academic researchers from the University of Antioquia, who sought to leverage the full electronic capacity of the Digital Hospital platform to compile robust clinical data registries for longitudinal health systems research. However, these metrics failed to achieve consensus due to consistently low scores from both the *promotoras de salud* and municipal decision-makers. The community health workers noted that complex, multi-step digital data entry protocols were fundamentally incompatible with the rapid, high-stress environment of mobile health screenings in an informal settlement, particularly when navigating geographical barriers or the immediate aftermath of local environmental disruptions. This observation is consistent with digital health implementations in fragile and unstable contexts, where environmental shocks and physical displacement necessitate minimalist, low-burden data inputs rather than complex clinical reporting structures (41).

Furthermore, this specific divergence mirrors documented “know-do” gaps in international health systems research, where high-level clinical experts frequently favor data-intensive electronic tracking systems, while frontline personnel rate those same strategies poorly due to immediate field-level feasibility constraints (Singh et al., 2023). This disconnect between epidemiological data desires and real-world workflow limits is a well-recognized barrier to technology adoption among community health networks globally (47). Municipal representatives converged with the health workers on this point, noting that the regional health infrastructure lacked the stable broadband capacity and hardware maintenance budgets required to sustain such intensive data streams, rendering the indicators an unsustainable administrative liability.

Conversely, a second area of non-consensus developed regarding the inclusion of informal, community-vetted traditional health practices and qualitative family narrative tracking within the pre-visit assessment framework. This strategy was highly valued by the community health workers, who recognized these relationship-based, culturally grounded insights as vital components for building patient trust and ensuring long-term community engagement with digital medicine. This perspective did not achieve consensus, however, because academic researchers and municipal decision-makers rated these elements as low-priority or methodologically unviable. Academic panelists expressed concerns regarding the standardization, scientific validity, and clinical reproducibility of informal narrative data within a digital health platform, mirroring broader literature on the historical marginalization of qualitative and traditional health indicators within formal, bio-centric clinical models (48).

Simultaneously, municipal representatives rejected these indicators due to strict data privacy regulations, legal frameworks governing formal medical records, and the administrative difficulty of aggregating non-standardized qualitative inputs into regional care coordination databases. Legal and regulatory compliance studies routinely demonstrate that privacy anxieties and rigid medical record legislation frequently stymie the integration of localized, non-standardized community health data into municipal systems (Kruse et al., 2018). This tension aligns precisely with contemporary Delphi research exploring global community health information systems, which highlights that while frontline health workers demand highly adaptable systems capable of capturing local contextual requirements, centralized administrative bodies routinely restrict tools to rigid frameworks to satisfy broader institutional interoperability and data-management standards (49). The exclusion of these items underscores a critical negotiation: balancing the desire for comprehensive data and institutional compliance against the absolute necessity of maintaining a streamlined, culturally acceptable, and field-feasible tool for frontline health workers.

### 4.4 Broader contributions to digital health evaluation

Taken together, the study's findings advance telehealth evaluation literature by expanding the empirical scope of what constitutes meaningful telehealth data, integrating social determinants, and institutionalizing community health worker involvement within the evaluation architecture. While these results provide a valuable methodological example of co-produced telehealth metrics, their application remains bounded by the exploratory and context-specific nature of this study. The Granizal framework can inform the theoretical underpinnings of digital health evaluation by demonstrating how participatory methods generate contextually valid measures that complement standardized global indicators (3, 47, 48). It demonstrates that participatory, consensus-based methods can generate contextually valid measures that complement and strengthen standardized global indicators and can improve healthcare delivery (49–51). This evaluation framework may be particularly relevant for humanitarian and displacement settings, where traditional evaluation tools often overlook community dynamics and informal care systems (2, 52, 53). By prioritizing community-defined indicators and iterative feedback mechanisms in the evaluation process, the approach aligns with recommendations from global bodies that call for adaptive digital health ecosystems that evolve with local contexts (38). Future validation in active implementation settings and across diverse geographic contexts remains a vital prerequisite before assuming the widespread transferability or external validity of the co-produced evaluation framework.

## 5 Conclusion

This Delphi study produced a community-informed telehealth evaluation framework that conceptualizes how social, infrastructural, and behavioral metrics might complement traditional clinical indicators in limited-resource settings. By operationalizing a consensus-building methodology with local stakeholders, the study offers an unvalidated, context-specific model designed to reflect the unique socio-environmental conditions of a single informal settlement. Rather than providing a finalized tool ready for widespread replication, this research establishes an exploratory foundation for future equity-focused evaluation design.

For health systems and administrative practitioners, the framework points to the conceptual value of participatory design, such as considering frontline health worker feedback and incorporating post-visit tracking parameters within localized care workflows. However, the real-world operationalization of these insights remains bounded by prevailing resource scarcity and institutional fragmentation. While historical regional examples suggest that community-driven digital health initiatives can function effectively under specific field conditions, the proposed evaluation architecture cannot be assumed to possess immediate operational feasibility until it undergoes rigorous empirical validation within active implementation settings.

Consequently, the primary value for researchers lies in the transferable nature of the participatory methodology rather than the universal applicability of the specific indicators generated by this panel. Because this framework was derived from an exploratory Delphi process in Granizal, Colombia, it is not generalizable and cannot be presumed transferable to other geographic contexts, urban peripheries, or displaced populations without systematic local adaptation and prospective validation. Future research must empirically test these co-produced indicators within real clinical workflows to verify their reliability, data-collection feasibility, and capacity to accurately capture health equity dynamics over time.

Ultimately, this study contributes to digital health literature by illustrating how local community perspectives can be systematically structured to challenge and expand conventional, centralized monitoring standards. By explicitly linking localized socioeconomic realities with broader technical requirements, this research proposes a conceptual pathway for designing telehealth evaluation systems that are intended to be socially inclusive. Confirming whether this framework can successfully bridge the gap between digital health aspirations and on-the-ground implementation remains dependent on future real-world validation.

## Data Availability

The raw data supporting the conclusions of this article will be made available by the authors, without undue reservation.
